# Stimulation of non-specific immunity, gene expression, and disease resistance in Nile Tilapia, *Oreochromis niloticus* (Linnaeus, 1758), by the methanolic extract of the marine macroalga, *Caulerpa scalpelliformis*

**DOI:** 10.14202/vetworld.2019.271-276

**Published:** 2019-02-16

**Authors:** Omita Yengkhom, Konda Subramanian Shalini, P. A. Subramani, R. Dinakaran Michael

**Affiliations:** Centre for Fish Immunology, Vels Institute of Science, Technology and Advanced Studies (VISTAS), Chennai, Tamil Nadu, India

**Keywords:** *Aeromonas hydrophila*, *Caulerpa scalpelliformis*, immunostimulant, macroalga, *Oreochromis niloticus*

## Abstract

**Aim:**

The objective of the present study was to test the immunostimulating potential of marine macroalga, *Caulerpa scalpelliformis*, in terms of non-specific immune responses, gene expression, and disease resistance of Nile tilapia, *Oreochromis niloticus* (Linnaeus, 1758).

**Materials and Methods:**

*O. niloticus* was injected intraperitoneally with three different doses of methanol extract of *C. scalpelliformis* (CSME) (2 mg/kg, 20 mg/kg, or 200 mg/kg body weight), or MacroGard™ (commercial immunostimulant, positive control, and 20 mg/kg body weight), or distilled water (untreated control). In one set of fish, 5 days post-injection, serum lysozyme, myeloperoxidase, and antiprotease activities were assayed. 24 h after injection, gene expression was analyzed in a separate set of fish. To another set of fish, 1 week post-administration of the products, fish were challenged with lethal dose 50 (LD_50_) dose of a live virulent pathogen, *Aeromonas hydrophila* and subsequent resistance to it was noted in terms of cumulative percent mortality.

**Results:**

CSME increased serum lysozyme, myeloperoxidase, and antiprotease activities. There was an increase in the expression of lysozyme gene in the spleen of treated fish. Mid dose of CSME caused the minimum mortality of 10% (consequent relative percentage survival = 73) which is comparable to that of the positive control.

**Conclusion:**

CSME is considered to have the potential to be developed into an immunostimulant for finfish aquaculture.

## Introduction

Fish are an excellent source of many vital nutrients including proteins, vitamins, and minerals required for the human body. Amount of fishes captured from oceans, seas, and rivers are diminishing rapidly. On the other hand, global aquaculture is expanding swiftly [1]. However, the substandard practices involved in fish rearing resulted in a range of fish diseases. Some of the disease treatment methods such as the use of antibiotics or vaccines cannot be successfully employed due to their limitations reviewed elsewhere [2]. Hence, the use of immunostimulants as a prophylactic measure for disease treatment is gaining much interest [3,4].

Macroalgae have various compounds with varied biological activities [5]. *Caulerpa scalpelliformis* belonging to Caulerpaceae family of macroalgae is one of the common seaweeds, found along the coast in many regions of the world. *Caulerpa* species have been shown to have antidiabetic [6], anti-inflammatory [7], antioxidant [8], and anti-proliferative [9] activities. Nile tilapia, *Oreochromis niloticus* also named as “Food fish of the 21^st^ century” [10] is robust fish which can dwell well even in hostile aquatic environments. *O. niloticus* is the second most reared fish after carps [11]. *Aeromonas hydrophila* is one of the most prevalent pathogens responsible for serious fish diseases such as motile aeromonad septicemia [12]. Hence, it is imperative to find out potential immunostimulants to fight against the onslaught of pathogens.

This study reports the modulation of non-specific immunity, expression of immune genes and protection from *A. hydrophila* infection in fish after an intraperitoneal administration of methanolic extract of *C. scalpelliformis* (CSME).

## Materials and Methods

### Ethical approval

The International Ethical Committee’s recommendation on “Guidelines for the use of fish in research” [13] was followed while handling the fish.

### Fish

All male-Nile tilapia (n=300), weighing 45±5 g were procured from Svara Biotechnovations, Madurai, India (10° 09” N, 78° 19” E). After acclimation to laboratory conditions for 2 weeks, experiments were conducted. All the experiments were conducted in 150 L fiber reinforced plastic tanks fitted with external re-circulating biofilters (Eheim, Deizisau, Germany). Fish were kept in ambient light and temperature (27±2°C) conditions throughout the experiments. Water quality parameters such as total dissolved solids, pH, and dissolved oxygen were maintained within permissible limits for Nile tilapia [11,14]. Ammonia and nitrate levels were minimized by the bacteria present in the biofilters.

### Preparation of methanol extract

The method of Das *et al*. [15] was followed for preparing CSME. Briefly, air-dried macroalgal material (150 g) was immersed in 2.5 L methanol and incubated at 15°C for 7 days with intermittent stirring. The resultant extract was filtered using a sterile muslin cloth, concentrated using a rotary vacuum evaporator (Buchi, Switzerland) and air dried to a pasty consistency. The dried extract was stored at −20°C till used for experiments.

### Experimental design

All the experiments were conducted according to our earlier procedures [14]. Each experiment consisted of five groups in triplicate tanks (total number of tanks = 15). To one group of fish, distilled water was injected that functioned as an untreated control group. The next three groups of fish were administered with three different doses of methanol extract 2, 20, or 200 mg/kg (body weight of fish). The last group was injected with 20 mg/kg MacroGard^™^ (Biotech Pharmacon ASA, Tromso, Norway) which is a commercially available immunostimulant and acted as a positive control.

### Serum non-specific immune assays

For assessing the non-specific immune mechanisms, six fish per group (treatments of different groups given above) were maintained in triplicate tanks and four fish from each tank were used (a total of 12 fish/group for each assay). Fish were first anesthetized in 100 ppm of 2-phenoxyethanol (HiMedia, Mumbai, India) followed by drawing 0.2 mL blood from the common cardinal vein [16] using a tuberculin syringe fitted with a 24 gauge needle. The collected blood in serological tubes was kept for 1 h at room temperature and further overnight at 4°C for separation of serum. Serum was then separated by spinning the serological tubes the next day for 10 min at 400× *g*. The serum obtained was stored at −20°C until used for the assays.

Serum lysozyme assay was done according to the method described earlier by Hutchinson and Manning [17] with *Micrococcus lysodeikticus* (Sigma-Aldrich, USA) as the substrate. The optical density (OD) was determined at 490 nm with a microplate reader (Bio-Rad, Hercules, USA). Reduction of OD by 0.001 absorbance unit per minute was defined as one unit of lysozyme activity [17].

Serum myeloperoxidase activity was measured using the protocol of Quade and Roth [18]. Briefly, sera were diluted 10-fold using phenol red-free Hank’s balanced salt solution having Mg^2+^ and EGTA. To the diluted sera, 50 µL tetramethylbenzidine-hydrogen peroxide (H_2_O_2_) (Genei, Bengaluru, India) was added and incubated for 2 min at room temperature for the color development. The color development reaction was abruptly stopped by the adding 50 µL 2M H_2_SO_4_. The OD of the samples versus 100 µL of HBSS as blank was noted at 450 nm.

The method of Bowden *et al*. [19] was used to measure the percentage inhibition of trypsin (serum antiprotease) activity. Serum (10 µL) was incubated with 0.1% trypsin (20 µL) (HiMedia, Mumbai, India) prepared in 0.01M Tris-HCl buffer, pH 8.2 for 5 min in serological tubes. The incubation was followed by the addition of 500 µL of sodium-benzoyl-DL-arginine-p-nitroanilide (BAPNA, SRL chemicals, Chennai, India) and 470 µL of buffer. Trypsin incubated with BAPNA alone was considered as trypsin blank for calculation. The incubation was continued for another 25 min at 22°C. Finally, the reaction was terminated by the addition of 30% acetic acid (150 µL). OD was measured at 415 nm by taking 200 µL of this reaction mixture against Tris-HCl buffer as blank. Percentage inhibition of trypsin activity was then calculated using the formula [14]:


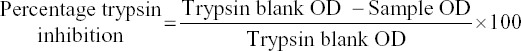


### Modulation of immune genes expression

For studying the expression of immune-associated genes, three fish per group (total 15 numbers) were distributed to five groups as mentioned above, and respective CSME products were intraperitoneally administered to the fish in each group. After 24 h of administration, fish were sacrificed by an overdose of 2-phenoxyethanol for the collection of the spleen. Collected spleens were preserved in RNAlater (Sigma-Aldrich, St. Louis, USA) and stored at −20°C until extraction of RNA was carried out. Gene expression analysis was then conducted according to our earlier protocols [14].

Total RNA was extracted using Trizol (Sigma, Bengaluru, India) following the manufacturer’s protocol. Subsequently, RNA extraction was followed by the synthesis of cDNA in a thermocycler (Eppendorf Mastercycler^®^ Nexus, Hamburg, Germany) with the use of Omniscript Reverse Transcription Kit (Qiagen, Bengaluru, India). To perform the gene expression analysis of lysozyme, β-actin was used as a housekeeping gene. REDTaq^®^ Readymix polymerase chain reaction (PCR) reaction (Sigma, Bengaluru, India) and nuclease-free water (Sigma, Bengaluru, India) including forward and reverse primers were used for the PCR reaction. The primer sequence and conditions for reactions are given in Table-1 [20,21]. Electrophoresis of the PCR products was performed in 1.5% agarose gel which included 10 µg/mL ethidium bromide (Medox, Chennai, India). Electrophoresed gel was then viewed in a gel documentation system (Proteinsimple, New Delhi, India) and photographed. The PCR product quantification was done with ImageJ v1.50b software for windows [22] using the photograph obtained from the gel documentation system.

**Table-1:** Details of primer sequences used in this study.

S.No.	Name of the gene	Annealing temperature	Primer sequences 3’→5’	PCR product size	References
1.	β-actin	60°C	F: CCACACAGTGCCCATCTACGA	100-110bp	[20]
			R: CCACGCTCTGTCAGGATCTTCA		
2.	TNF-α	55°C	F: CCTGGCTGTAGACGAAGT	134bp	[21]
			R: TAGAAGGCAGCGACTCAA		
3.	Lysozyme	60°C	F: TTGGGAGTGTTCAACAGTGG	300bp	Self-designed (http://primer3.ut.ee/)
			R: GCCTCTGACAGCATTTGACA		

PCR=Polymerase chain reaction

### Challenge to fish

Fish in the five different groups (10 fish/group in triplicates) as mentioned above were injected intraperitoneally with various doses of CSME, MacroGard™ or sterile distilled water. 1 week post-injection, fish in all the five groups were challenged with lethal dose 50 (LD_50_) of *A. hydrophila*. Cumulative mortality for a period of 15 days was noted and used for calculating the percent mortality. *A. hydrophila* was reisolated from the tissues of dead fish using Rimler-Shott’s medium (HiMedia, Mumbai, India) to confirm the death of the fish was due to *A. hydrophila*. Relative percent survival was then calculated from percentage mortality using the formula used in our previous studies [14]:


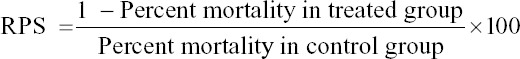


### Statistical analysis

Statistical analysis was done with the use of Sigmaplot v.11. Data were expressed as mean ± standard error and analyzed by one-way ANOVA followed by Tukey’s a posteriori test with a significance level of p<0.05.

## Results

**Figure-1:**
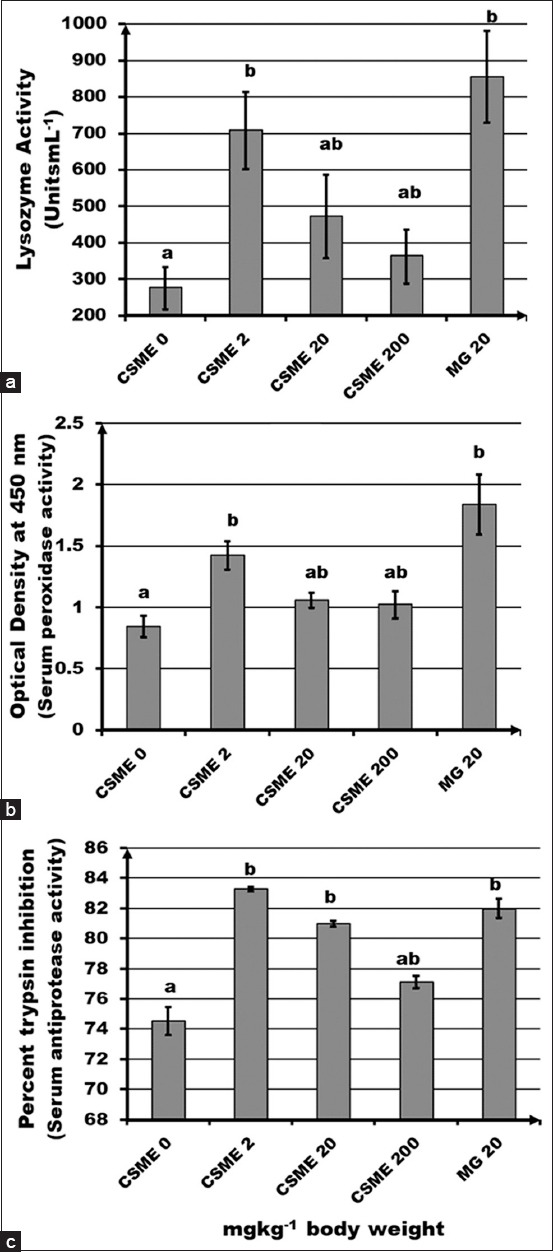
Effect of intraperitoneal administration (mg/kg body weight) of *Caulerpa scalpelliformis* of methanolic extract on (a) serum lysozyme, (b) serum myeloperoxidase, and (c) serum antiprotease activities. Each point represents mean ± standard error of 12 fish. Different alphabets represent significant difference (p<0.05) between means as assessed by one-way ANOVA with Tukey’s a posteriori test.

The low dose of CSME boosted the serum lysozyme activity which was significantly (p<0.05) different from that of the untreated control. MacroGard™ also significantly increased lysozyme activity (Figure-1a). Enhancement in serum myeloperoxidase by a low dose of CSME was significantly (p<0.05) different from that of the untreated control and its effect was indifferent to that of MacroGard™ (Figure-1b). In serum antiprotease activity, the low and mid doses of CSME significantly (p<0.05) enhanced the percent trypsin inhibition comparable to that of the positive control, MacroGard™ (Figure-1c).

**Figure-2:**
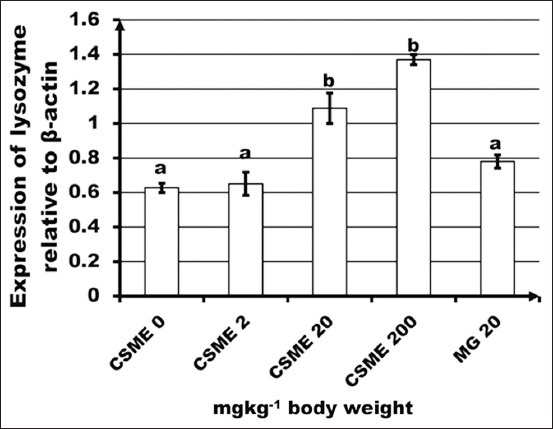
Effect of *Caulerpa scalpelliformis* methanolic extract on lysozyme expression in the spleen of *Oreochromis niloticus*. Each point represents mean ± standard error of 3 fish. Different alphabets represent a significant difference (p<0.05) between means as assessed by one-way ANOVA with Tukey’s a posteriori test.

**Figure-3:**
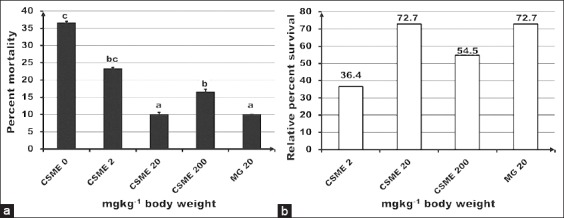
Effect of *Caulerpa scalpelliformis* methanolic extract on disease resistance in terms of (a) percent mortality to *Aeromonas hydrophila* and (b) relative percent survival. Each point represents mean ± standard error of 30 fish. Different alphabets represent a significant difference (p<0.05) between means as assessed by one-way ANOVA with Tukey’s a posteriori test.

The low and high doses of CSME caused significant (p<0.05) upregulation of lysozyme gene expression though positive control did not show any enhancement (Figure-2). The mid and high doses of CSME and positive control significantly reduced the percent mortality of fish infected with *A. hydrophila*. As shown in Figure-3, the mid dose and MacroGard™ showed the lowest percentage mortality (10%).

## Discussion

There is an urgent requirement for alternatives to vaccines and antibiotics for disease control in aquaculture. Plant-derived immunostimulants have been found to improve the non-specific immunity in fish by enhancing the immune mechanisms such as lysozyme, myeloperoxidase, and expression of immune genes [23,24]. The current study explores the potential of CSME in boosting the non-specific immunity in *O. niloticus* with the subsequent protection from *A. hydrophila* infection. The quaternary alkaloids and polysaccharides fractions from the same macroalga had been reported earlier to be immunostimulating in the striped murrel, *Channa striata*, and *O. niloticus*, respectively [14,25].

Lysozyme is considered as a crucial bactericidal enzyme of the innate immunity [26]. Mice lacking lysozyme showed a decline in the removal of *Pseudomonas aeruginosa* from the airways [27]. In the present study, treatment with a low dose of CSME to *O. niloticus* resulted in augmentation of the lysozyme activity when compared with that of the untreated control. In comparable studies conducted elsewhere, Mozambique tilapia fed with a diet enriched with ethanolic extract of bee glue, Propolis, showed an increase in the serum lysozyme activity [23]. Polysaccharide from the brown alga *Padina gymnospora* also boosted the serum lysozyme activity in carps [28]. Increased lysozyme activity protects fishes from various pathogens and confers a higher degree of disease resistance [29]. In the present investigation, the mid and high doses of CSME increased the expression of gene encoding lysozyme. Recently in the same fish species, the plant-derived traditional Chinese medicine preparation has been shown to enhance the expression of lysozyme gene [30].

Myeloperoxidase produced in the neutrophils catalyzes the formation of hypochlorous acid from hydrogen peroxide which is lethal to living cells [31]. Infection with *Candida albicans* in myeloperoxidase lacking zebrafish led to a failure in the removal of the pathogen [32]. In the present study, the low dose resulted in an increase in myeloperoxidase activity. Supplementation of *Ecklonia cava*, a brown alga to the diet of olive flounder, increased the myeloperoxidase activity [33]. In another similar study, the diet of kelp grouper, *Epinephelus bruneus*, was enriched with ethanol extract of *Siegesbeckia glabrescens* that augmented the production of myeloperoxidase [34].

Antiprotease acting against the proteolytic enzymes secreted by pathogens also has a role in acute phase reactions [35]. In the present study, a significant boost in antiprotease activity was noticeable by the action of low and mid doses. This is in agreement with another finding in this laboratory wherein intraperitoneal administration of methanolic extract of night jasmine seeds in tilapia increased the serum antiprotease activity [36]. *Sophora flavescens* as a dietary supplement in tilapia also led to an increase in the antiprotease activity [37].

Challenging fish with an LD of the microbe and checking its survival can be used as a “golden standard” to determine the potential of an immunostimulant [38]. Here, in this study, the mid and high doses conferred very good protection in fish against *A. hydrophila* when compared to the untreated control. African catfish fed with diet supplemented with ethanolic extract of *Ocimum gratissimum* maximized protection from *Listeria monocytogenes* [39]. Survival in tilapia against *Streptococcus agalactiae* was increased when fed for 20 days with a diet supplemented with aqueous extract of *Cratoxylum formosum* [40]. Methanolic extract of *Punica granatum* leaves enhanced resistance in olive flounder from lymphocystis disease virus [41].

## Conclusion

Intraperitoneal administration of CSME stimulated the non-specific immune responses of *O. niloticus*. CSME protected *O. niloticus* when infected experimentally with *A. hydrophila*. Hence, CSME used in the study has the potential of being a candidate for immunostimulant in finfish aquaculture. However, field trials will be a prerequisite before any application attempt in large-scale farms.

## Authors’ Contributions

The results reported herewith are part of OY’s doctoral thesis. RDM designed the work. OY and KSS did the experiments and collected the results. PAS and RDM compiled the results and drafted the manuscript. All authors read and approved the final manuscript.
